# Phosphomimetic Mutation of Cysteine String Protein-α Increases the Rate of Regulated Exocytosis by Modulating Fusion Pore Dynamics in PC12 Cells

**DOI:** 10.1371/journal.pone.0099180

**Published:** 2014-06-23

**Authors:** Ning Chiang, Yu-Tien Hsiao, Hui-Ju Yang, Yu-Chun Lin, Juu-Chin Lu, Chih-Tien Wang

**Affiliations:** 1 Institute of Molecular and Cellular Biology, National Taiwan University, Taipei, Taiwan; 2 Department of Life Science, National Taiwan University, Taipei, Taiwan; 3 Neurobiology and Cognitive Science Center, National Taiwan University, Taipei, Taiwan; 4 Genome and Systems Biology Degree Program, National Taiwan University, Taipei, Taiwan; 5 Department of Physiology and Pharmacology, College of Medicine, Chang Gung University, Tao-Yuan, Taiwan; UPR 3212 CNRS -Université de Strasbourg, France

## Abstract

**Background:**

Cysteine string protein-α (CSPα) is a chaperone to ensure protein folding. Loss of CSPα function associates with many neurological diseases. However, its function in modulating regulated exocytosis remains elusive. Although *cspα*-knockouts exhibit impaired synaptic transmission, overexpression of CSPα in neuroendocrine cells inhibits secretion. These seemingly conflicting results lead to a hypothesis that CSPα may undergo a modification that switches its function in regulating neurotransmitter and hormone secretion. Previous studies implied that CSPα undergoes phosphorylation at Ser^10^ that may influence exocytosis by altering fusion pore dynamics. However, direct evidence is missing up to date.

**Methodology/Principal Findings:**

Using amperometry, we investigated how phosphorylation at Ser^10^ of CSPα (CSPα-Ser^10^) modulates regulated exocytosis and if this modulation involves regulating a specific kinetic step of fusion pore dynamics. The real-time exocytosis of single vesicles was detected in PC12 cells overexpressing control vector, wild-type CSPα (WT), the CSPα phosphodeficient mutant (S10A), or the CSPα phosphomimetic mutants (S10D and S10E). The shapes of amperometric signals were used to distinguish the full-fusion events (i.e., prespike feet followed by spikes) and the kiss-and-run events (i.e., square-shaped flickers). We found that the secretion rate was significantly increased in cells overexpressing S10D or S10E compared to WT or S10A. Further analysis showed that overexpression of S10D or S10E prolonged fusion pore lifetime compared to WT or S10A. The fraction of kiss-and-run events was significantly lower but the frequency of full-fusion events was higher in cells overexpressing S10D or S10E compared to WT or S10A. Advanced kinetic analysis suggests that overexpression of S10D or S10E may stabilize open fusion pores mainly by inhibiting them from closing.

**Conclusions/Significance:**

CSPα may modulate fusion pore dynamics in a phosphorylation-dependent manner. Therefore, through changing its phosphorylated state influenced by diverse cellular signalings, CSPα may have a great capacity to modulate the rate of regulated exocytosis.

## Introduction

Secretion of neurotransmitters and hormones from vesicles is mediated by regulated exocytosis. During the initial stage of vesicle fusion, the fusion pore is a dynamic structure that transiently connects the vesicle to the plasma membrane, and subsequently undergoes full dilation to cause membrane fusion [1]. The minimal fusion machinery is formed by the four α-helical bundles in the soluble N-ethylmaleimide-sensitive factor attachment protein receptor (SNARE) complex. This complex comprises two t-SNARE proteins, syntaxin (Syx) and synaptosome-associated protein of 25 kDa (SNAP-25/SN25), on the plasma membrane, and one v-SNARE protein, synaptobrevin (Syb), on the vesicle membrane. This complex and its constituents can interact with the Ca^2+^ sensor protein synaptotagmin (Syt) [2]. Upon Ca^2+^ entry, Syt regulates fusion pore opening and dilation, through binding to Ca^2+^ molecules and triggering the downstream interactions, resulting in vesicle fusion and transmitter release [2], [3], [4], [5], [6], [7], [8], [9], [10], [11]. Recently, several studies have identified a co-chaperone cysteine string protein (CSP) that facilitates the correct folding of SNARE proteins, e.g., Syb [12], SN25 [13], and SNARE complex [14]. Thus, CSP may function as a modulator of the final fusion process during regulated exocytosis [15], [16].

CSP, first discovered in *Drosophila* [17], is a vesicle membrane protein localized to both synaptic vesicles (SVs) [18] and large dense-core vesicles (LDCVs) [19], [20], [21]. Three CSP isoforms (α, β, and γ) have been found in mammal, but only CSPα is expressed in brain and endocrine cells [22]. Deletion of CSPα in *Drosophila* or mouse impairs synaptic transmission, leading to progressive neurodegeneration and premature death [22], [23], [24]. In addition, suppression of CSPα expression reduces Ca^2+^-dependent LDCV exocytosis in permeabilized pancreatic β-cells [25]. These two results suggest that CSPα promotes neurotransmitter release. However, overexpression of CSPα also inhibits secretion in pancreatic β-cells [20] and adrenal chromaffin cells [21], implying that CSPα inhibits regulated exocytosis. These seemingly conflicting results have led to the hypothesis that CSPα may undergo a modification at certain condition that switches its function in regulating neurotransmitter and hormone secretion. Interestingly, in neuroendocrine cells and mammalian brain, the Ser^10^ residue at CSPα (CSPα-Ser^10^) can be phosphorylated by both protein kinase A (PKA) and protein kinase B (PKB)/Akt [15], [26], [27], [28]. Thus, this specific serine residue may serve as a site where cellular signals converge to modulate the function of CSPα in regulated exocytosis. Further studies showed that phosphorylation of CSPα-Ser^10^ limits the interaction between CSPα and Syt isoforms, e.g., Syt I and IX [15], [16], suggesting that CSPα phosphorylation may regulate fusion pore dynamics. In addition, CSPα regulates the polymerization of dynamin I, a protein mediating vesicle fission, suggesting that CSPα participates in synaptic vesicle endocytosis [29]. However, direct evidence is currently missing whether phosphorylation of CSPα-Ser^10^ affects fusion pore dynamics during regulated exocytosis.

Here, we addressed this question using amperometry, an electrochemical technique with high temporal resolution. This technique can resolve the dynamics of fusion pores created by individual vesicles containing readily oxidizable neurotransmitters, e.g., norepinephrine (NE). For a vesicle undergoing full fusion, amperometric recordings indicate the initial opening of a fusion pore as a prespike foot (PSF) that reflects the vesicle content leaking out through the fusion pore [30]. This transient fusion pore can either dilate to expel all the content, producing a spike in amperometric recordings, or it can close and retain most of the content, producing the kiss-and-run event owing to transient fusion without full dilation [31], [32]. Given that spikes preceded by prespike feet are established as full-fusion events, whereas square-shaped, stand-alone flickers are established as kiss-and-run events [30], [31], [32], [33], [34], the amperometric shape has been widely used to reveal various molecular mechanisms controlling fusion pore dynamics in the past decade [3], [6], [8], [35], [36], [37], [38], [39]. In this study, we combined single-event amperometry with molecular perturbation to investigate how the phosphomutants of CSPα-Ser^10^ modulate regulated exocytosis and if this modulation involves regulating a specific kinetic step of fusion pore dynamics.

## Results

### CSPα phosphodeficient mutation decreases the secretion rate from PC12 cells

Three isoforms of CSP (α, β, and γ) have been reported previously [22]. Thus, we used RT-qPCR to determine the major isoform(s) expressed in PC12 cells (Text S1). We found that the mRNA expression level of CSPα was significantly higher than that of either CSPβ (*p*<0.05) or CSPγ (*p*<0.05), suggesting that CSPα is likely the most abundant isoform expressed in PC12 cells (Figure S1-A). CSPα exists in two RNA splicing variants, CSPα1 and CSPα2 [20]. We further studied the cDNA of PC12 cells and confirmed that CSPα1, but not CSPα2, exists in PC12 cells (Figure S1-A, inset). Thus, in all our experiments, we molecularly perturbed CSPα1, and from here on, we will refer to CSPα1 simply as CSPα.

To study how phosphorylation affects CSPα's function, we used site-directed mutagenesis to create one phosphodeficient mutant (S10A) and two phosphomimetic mutants (S10D and S10E) of CSPα. We transfected the control vector pIRES2EGFP (Ctrl), wild-type CSPα (WT), or phosphomutant CSPα (S10A, S10D, or S10E) into PC12 cells by electroporation, allowing overexpression after 3 days post transfection for further experiments. To confirm the effectiveness of transfection, we examined the mRNA expression levels of different CSP isoforms in these transfected groups. In all CSPα-transfected groups, the mRNA levels of CSPα were 20-fold more than those levels of CSPβ or CSPγ (Figure S1-B). Western analysis also confirmed that CSP was overexpressed more in all CSPα-transfected groups compared to Ctrl (Text S1 and Figure S1-C).

**Figure 1:**
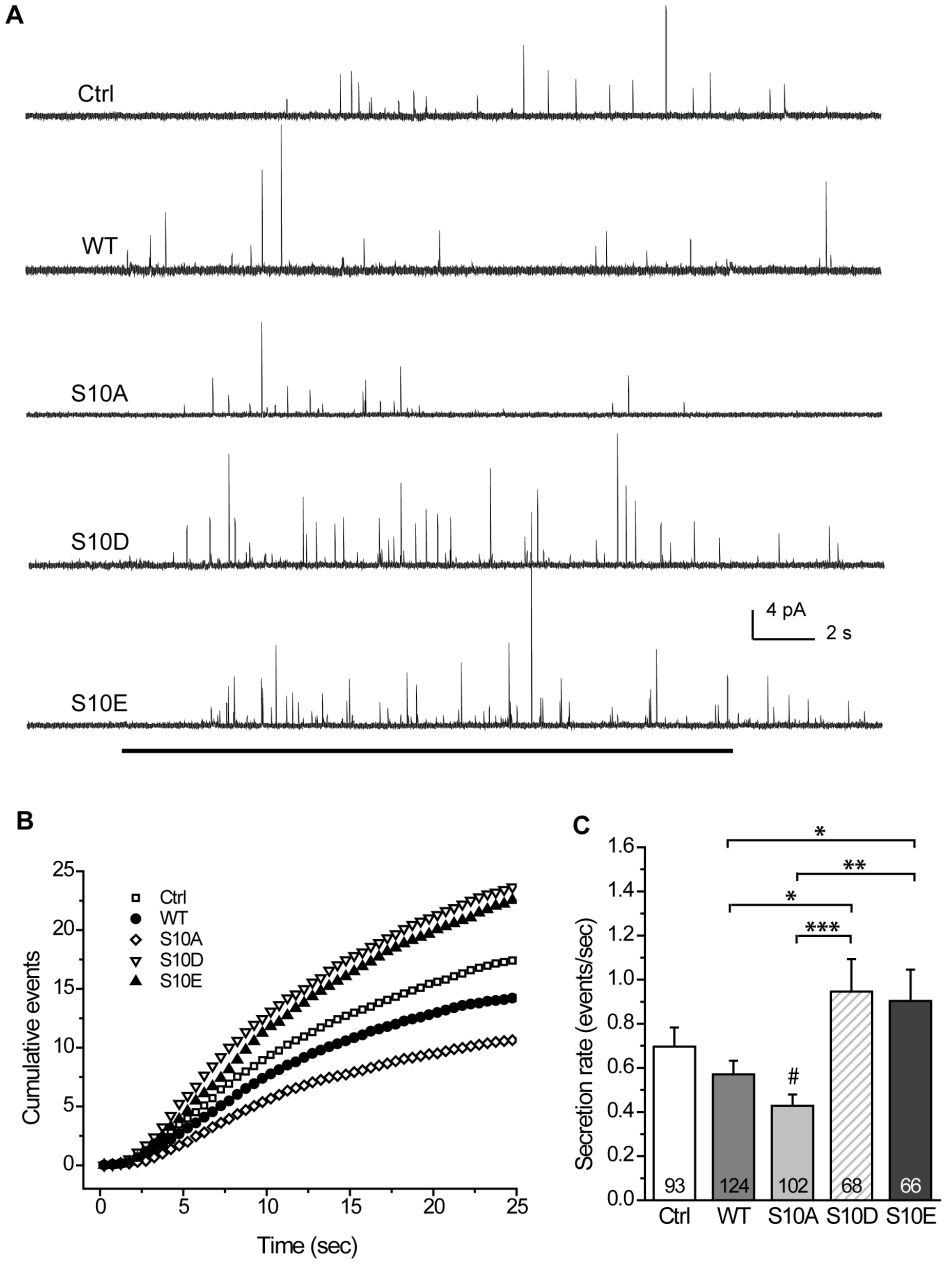
Secretion events in PC12 cells that overexpress CSP and its phosphomutants. **A**, Representative amperometric recordings of secretion events in PC12 cells that overexpress control vector (Ctrl), wild-type (WT), S10A, S10D, or S10E CSPα. Black line at bottom indicates the duration of KCl application (20 sec). **B**, Cumulative events for groups described in (A). Cumulative counts were normalized by cell numbers. **C**, The secretion rate was acquired from the onset of KCl application to the end of the recording (total 25 sec) by using the cellular mean method. Numbers in bars indicate number of cells recorded for each group. Total 1092–1771 events for those cells.

To determine if this overexpression affected the secretion rate, we performed single-event amperometry to detect Ca^2+^-dependent NE release from transfected cells in each group. We recorded amperometric traces by applying KCl to depolarize the cells and trigger Ca^2+^-regulated exocytosis (Fig. 1A). Plots of the cumulative fusion events (for events with peak amplitude ≥2 pA) [3] showed the different secretion rates across the various groups (Fig. 1B). We compared the rate of fusion events ≥2 pA (or secretion rate) for individual PC12 cells across the groups (Fig. 1C). We found that wild-type CSPα overexpression (from here on termed WT) gave the secretion rate comparable to overexpression of control vector (termed Ctrl). Moreover, S10A overexpression (termed S10A) produced the secretion rate comparable to WT, but significantly lower (*p*<0.05) compared to Ctrl (Fig. 1C). However, overexpression of the CSPα phosphomimetic mutants significantly altered the rates. S10D overexpression (termed S10D) evoked the secretion rate that was significantly higher than WT (*p*<0.05) or S10A (*p*<0.001). Similarly, S10E overexpression (termed S10E) gave the secretion rate that was significantly higher than WT (*p*<0.05) or S10A (*p*<0.01). These findings suggest that phosphodeficiency in CSPα-Ser^10^ may decrease the secretion rate during regulated exocytosis.

### CSPα phosphomutation does not change the expression levels of essential exocytotic proteins, or the targeting of CSPα to cell periphery

Since CSPα serves as a chaperone to ensure correct folding and thus can stabilize exocytotic proteins [12], [13], [14], we next examined if CSPα phosphorylation affects the expression levels of the Ca^2+^ sensor Syt I or of the SNARE proteins (Syx I, SN25, and Syb). We found that overexpression of CSPα or its phosphomutants did not affect the levels of any of these proteins (Text S1 and Figure S1-C), suggesting that CSPα phosphomutation does not alter the expression levels of essential exocytotic proteins.

Since we are studying release from LDCVs, we next checked whether CSP localized to cell periphery after KCl depolarization in PC12 cells overexpressing CSPα or its phosphomutants (Text S1 and Figure S1-D). We found that CSP mainly localized to cell periphery in cells overexpressing CSPα and its phosphomutants (Figure S1-D). Hence, overexpression of CSPα or its phosphomutants may not alter the subcellular localization of CSPα.

### CSPα phosphomimetic mutation prolongs the opening of the initial fusion pore that forms before dilation

**Figure 2:**
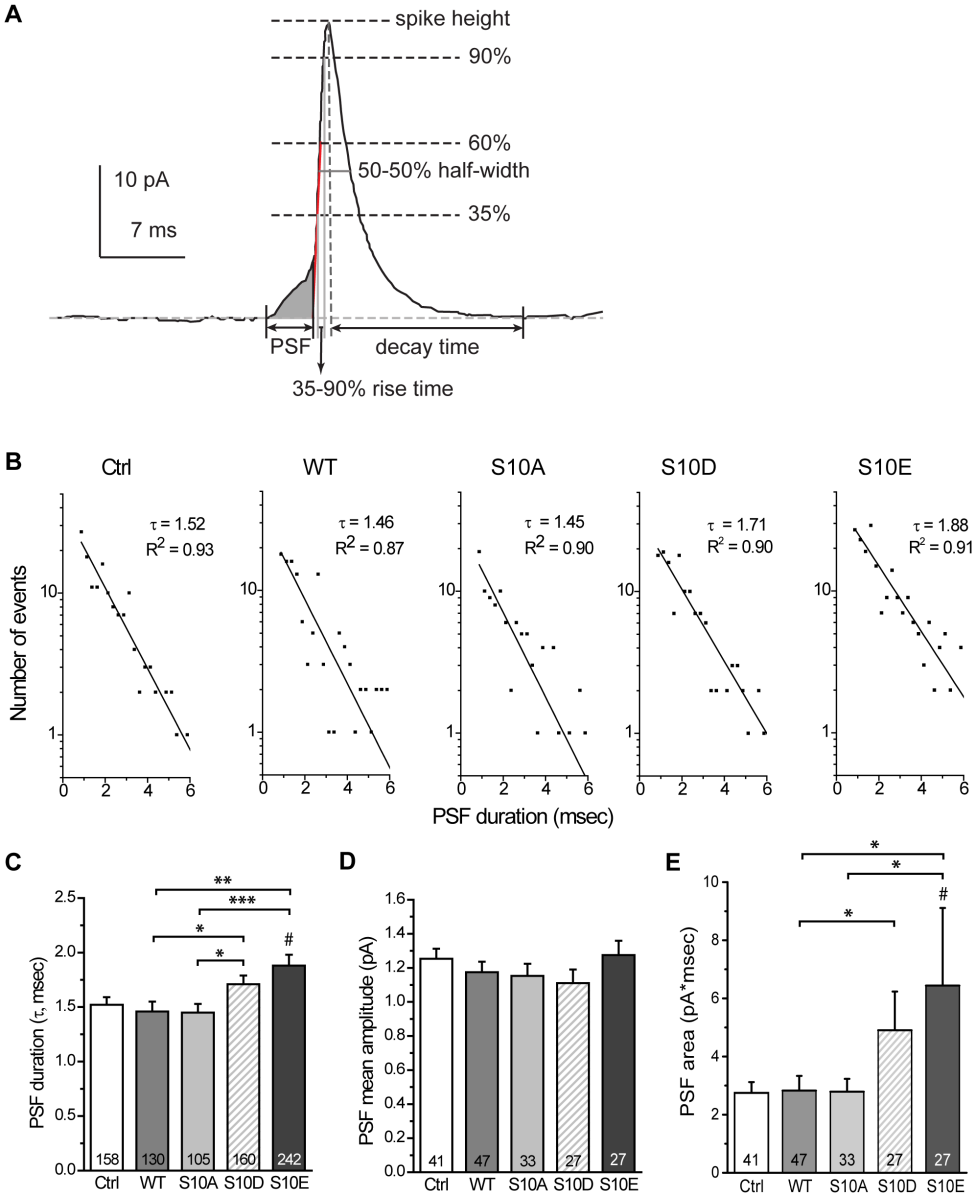
Characteristics of the PSF in cells overexpressing CSP and its phosphomutants. **A**, The amperometric recording for a full-fusion event from a single vesicle. The prespike foot (PSF, shown as shaded area) reports the initial opening of a fusion pore and the spike begins when the fusion pore enters into the dilation phase. The PSF lifetime was measured from onset (the current rising to 1×RMS noise above the baseline current) to end point (the intersection between the baseline and the red line going through the rise phase from 35 to 60% of the peak). The features of PSF and spikes analyzed in this study are illustrated (see Materials and Methods for detail). **B**, PSF lifetime distributions were constructed for the indicated groups. Distributions were fitted by the single-exponential decay function N(t) = N(0)×exp (−t/τ) to yield the mean PSF duration τ (the goodness of the fits: R^2^∼0.90). **C**, τ obtained in (B) for each group. Numbers in bars indicate the number of PSF used for each calculation. **D**, PSF mean amplitudes calculated by the cellular means method. **E**, PSF areas calculated by the cellular means method. For D and E, numbers in bars indicate the number of cells used for each calculation.

In amperometric recordings, the small rising signal that precedes a steep spike is referred to as a PSF, and it represents the transient opening of the initial fusion pore prior to dilation (Fig. 2A, shaded area). To examine if CSPα phosphomutation affects the opening of this initial fusion pore, we analyzed the characteristics of the PSF associated with spikes. For each transfection group, we constructed the histograms of PSF lifetimes in a semi-logarithmic plot (termed PSF lifetime distributions) and fitted the data by a single-exponential decay function (Fig. 2B) [3], [6]. The fitted lines gave the mean PSF duration, τ (Fig. 2B and C). The WT group's τ was similar to that for Ctrl and S10A groups. The S10D and S10E groups had comparable τ's but both of these were higher than those for other groups: the S10D group's τ was significantly higher than that for S10A (*p*<0.05) and WT (*p*<0.05) groups; the S10E's τ was significantly higher than that for S10A (*p*<0.001), WT (*p*<0.01), and Ctrl (*p*<0.05) groups. These results suggest that CSPα phosphomimetic mutation prolongs the opening of the fusion pore that leads to dilation. Notably, the mean amplitude of the PSF showed no significant difference across groups (Fig. 2D), suggesting that CSPα phosphomutation does not affect the flux through the initial fusion pore. In comparison, the PSF area was significantly increased by phosphomimetic mutants: the S10D group's PSF area was significantly higher than that for WT (*p*<0.05) groups; the S10E's PSF area was significantly higher than that for S10A (*p*<0.05), WT (*p*<0.05), and Ctrl (*p*<0.05) groups (Fig. 2E). Thus, CSPα phosphomimetic mutation may alter the kinetics of the initial fusion pore that forms prior to dilation.

### CSPα phosphodeficient mutation increases the fraction of kiss-and-run events

**Figure 3:**
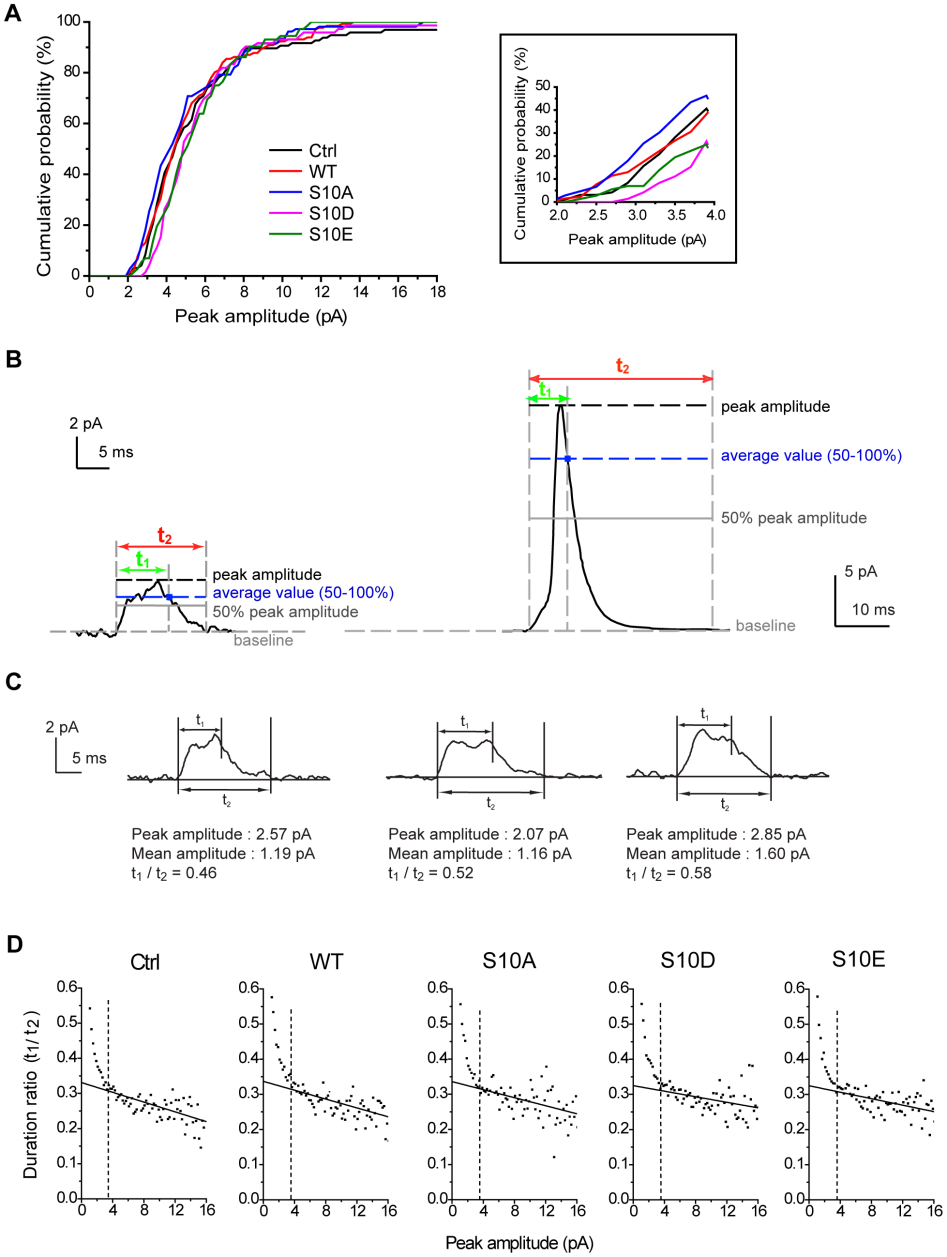
Two types of fusion events in cells overexpressing CSP and its phosphomutants. **A**, Cumulative probability of peak amplitudes for all events ≥2 pA. Inset, expanded view of this cumulative probability in the 2 to 4 pA amplitude range. **B**, Representative traces of kiss-and-run (KR) (left) and full-fusion (right) events. Two durations distinguish KR from full-fusion events. Duration t_1_ (green) represents the duration from signal onset to when the signal falls back to the average value above the 50% peak amplitude. Duration t_2_ (red) represents the duration from onset to when the signal returns to within 1×RMS noise of the baseline. Black dashed lines, the peak amplitude of the event. Gray solid lines, 50% of peak amplitude. Blue dashed lines, the average value of the data within 50–100% peak amplitude. Gray dashed lines, baseline (horizontal) or the time boundaries (vertical) for duration t_1_ or t_2_. Blue squares, the end point of duration t_1_. **C**, Peak amplitudes, mean amplitudes, and duration ratios can be obtained from individual KR events. **D**, Scatter plots of duration ratios (t_1_/t_2_) vs. peak amplitudes for events in all groups (*n* = 5583–9198 events). Duration ratios were averaged for events with peak amplitudes in the same bin and plotted versus peak amplitude (Note that we acquired all signals with the peak amplitude ≥1 pA here). Solid lines indicate the linear fits for the events with peak amplitude >3.5 pA. Dashed lines indicate the transition point (3.5 pA of peak amplitude) between the solid lines and the rising phase of the data.

Since CSPα phosphomimetic mutation can regulate the kinetics of the fusion pore that lead to full dilation, we next investigated if CSPα phosphomutation alters the dynamics of transient fusion without full dilation, i.e., kiss-and-run (termed KR). To study these KR events, we collectively examined all events that we defined as signals (peak amplitude ≥2 pA) and constructed the cumulative probability of the peak amplitudes for all events (Fig. 3A). Compared to Ctrl and WT, the half-maximal probability right-shifted for the S10D and S10E groups, and left-shifted for the S10A group. These tendencies were most apparent in events with peak amplitude between 2–4 pA (Fig. 3A, inset). In the zoomed traces of amperometric recordings (Figure S2), we found that most of these small events were of square-like shape, representing putative KR events [30], [31], [32], [33], [34]. Thus, the right shifts in the cumulative probability of the peak amplitudes for S10D and S10E suggest that phosphomimetic mutants exhibit fewer numbers of KR events.

To further explore if CSPα phosphorylation plays a role in regulating KR events, we utilized an approach described in a previous study [6] to analyze these two types of fusion events in our amperometric recordings: KR events (i.e., square-like events) (Fig. 3B, left) and full-fusion events (i.e., PSF followed by spikes) (Fig. 3B, right). To distinguish these events, we examined two values of duration, t_1_ and t_2_ (Fig. 3B). Duration t_1_ indicates the time between the onset of the rise and when the signal falls back to the average value of amplitude above the 50% peak amplitude. Duration t_2_ represents the time between the onset of the rise and when the signal returns to baseline [6] (see Materials and Methods). We found that the ratio of t_1_ to t_2_ was very sensitive to the event shape. Square-like events exhibit larger t_1_/t_2_ ratios than spike-like events (Fig. 3B–C). Thus, the ratio of t_1_ to t_2_ provided an index of event shape that was used to evaluate the putative KR events or spikes.

Scatter plots showed the relationship between peak amplitudes and t_1_/t_2_ ratios for all events in the transfected groups (Fig. 3D). In this analysis, the ratios of t_1_ to t_2_ were averaged for the events with peak amplitudes in the same bin and plotted versus peak amplitudes [6]. We found that the ratios (t_1_/t_2_) for peak amplitudes ≥3.5 pA had a steady but low value and can be fitted by linear regression (Fig. 3D, solid lines). By contrast, the ratios (t_1_/t_2_) started to rise sharply and shift apart from the fitting lines at 3.5 pA. This plot thus demonstrates a difference in shape between spikes and KR events, with a transition point just below 3.5 pA of peak amplitude. Thus, the cut-off peak amplitudes of 3.5 pA (Fig. 3D, dashed lines) can be used to divide the whole population of events into spike-like (full fusion) events and square-like (considered as KR) events.

To further verify whether these putative KR events (i.e., signals with peak amplitudes of 2–3.5 pA) are square-like events, we constructed their “*mean*” amplitude distributions for these square-like events. The mean amplitude was calculated as event area divided by duration, for comparison with PSF mean amplitude (see Materials and Methods). The putative KR events should confer two characteristics in their mean amplitudes. First, the mean amplitudes of putative KR events should be indistinguishable from those of PSF because both of them are generated from the non-dilating fusion pore. Second, in contrast to spikes where the dilating fusion pore allows the NE flux to rise sharply, the NE flux does not increase as the pore stays open in the putative KR events.

**Figure 4:**
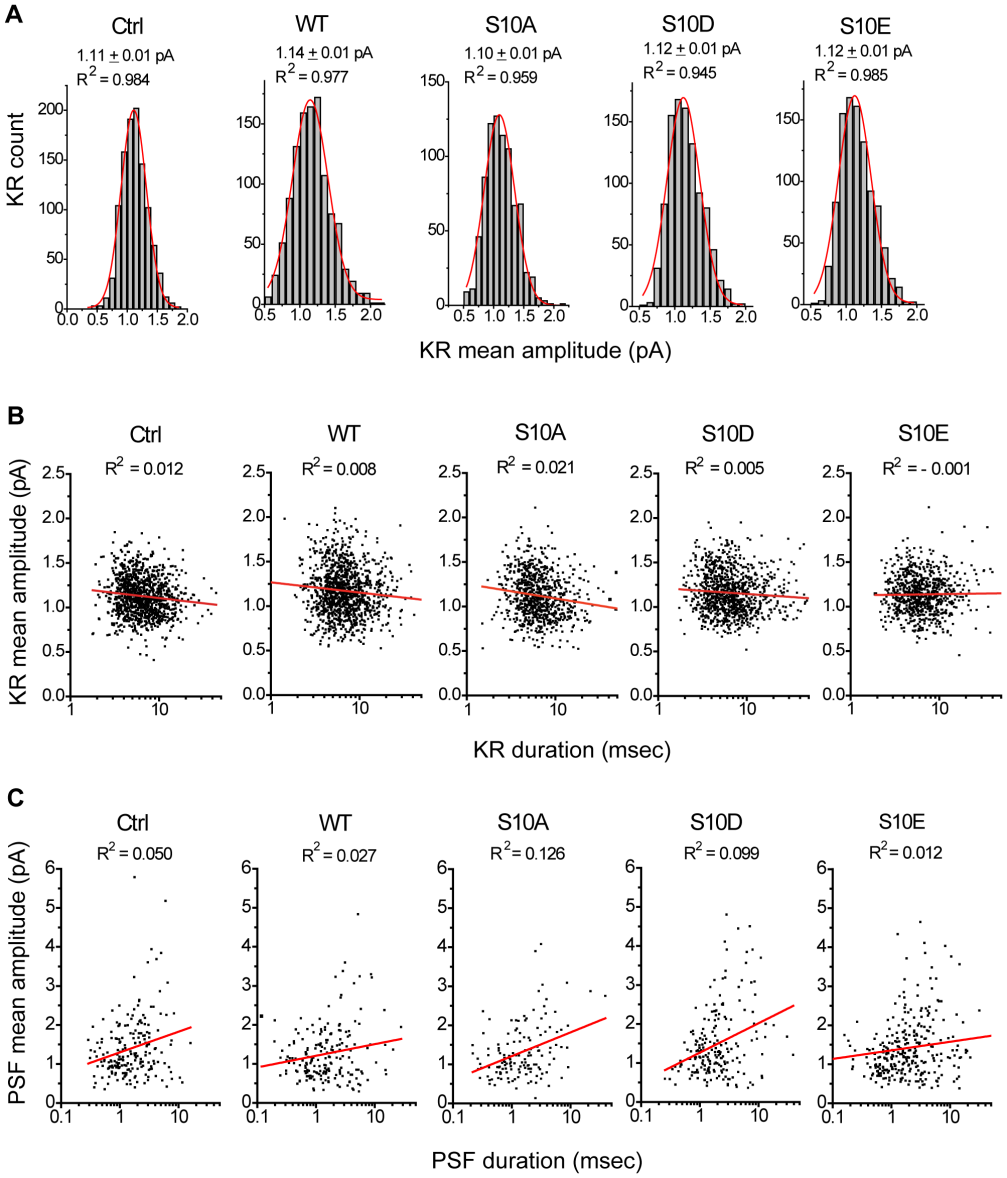
KR events in cells overexpressing CSP and its phosphomutants. **A**, Mean amplitude histograms of KR events fitted by Gaussian distributions (red) for all groups. The mean values and R^2^ from Gaussian distributions were as indicated. **B**, Scatter plots of mean amplitudes vs. durations for KR events. The R^2^ from linear regression (red) were as indicated. For A and B, *n* = 797–1112 events. **C**, Scatter plots of PSF mean amplitudes vs. durations. The R^2^ from linear regression (red) were as indicated. Total 105–242 PSF events.

We found that in all groups, these mean amplitude distributions for putative KR events (the signals with peak amplitudes of 2–3.5 pA corresponding to mean amplitudes of 0.3–2 pA) can be fitted by a single Gaussian distribution (Fig. 4A), suggesting that these square-like events arise from a single distinct population as reported previously [6]. Moreover, the mean amplitude of putative KR events (Fig. 4A) were comparable to those of PSF (Fig. 2D), suggesting that the KR events were from the non-dilating fusion pore. In contrast, the mean amplitude distributions for the events ≥3.5 pA were relatively skewed (the signals with peak amplitudes ≥3.5 pA corresponding to mean amplitudes >1 pA) (Figure S3), similar to the previous reports for spike height or area [6], [40], [41], [42], [43].

Since KR events confer the unique square or rectangular shape [31], [32], we determined if the shape of these 2–3.5 pA events is essentially square (or rectangular). We used a scatter plot to examine the correlation between the duration and mean amplitude of these events (Fig. 4B). Linear fitting of these scatter plots showed little correlation (R^2^ ranging from −0.001 to 0.021), suggesting that these 2–3.5 pA events seem to be square- or rectangular-shaped signals whose amplitudes do not change over time, corresponding to the opening of non-dilating fusion pores [6], [31], [32], [33], [34], [35]. Similarly, we found little correlation between the PSF's durations and mean amplitudes (R^2^ = 0.012–0.126) (Fig. 4C). Thus, the 2–3.5 pA events reflect KR events, corresponding to the transient openings of initial fusion pores. In some cases, these fusion pores dilate and lead to full fusion, producing PSF followed by spikes.

**Figure 5:**
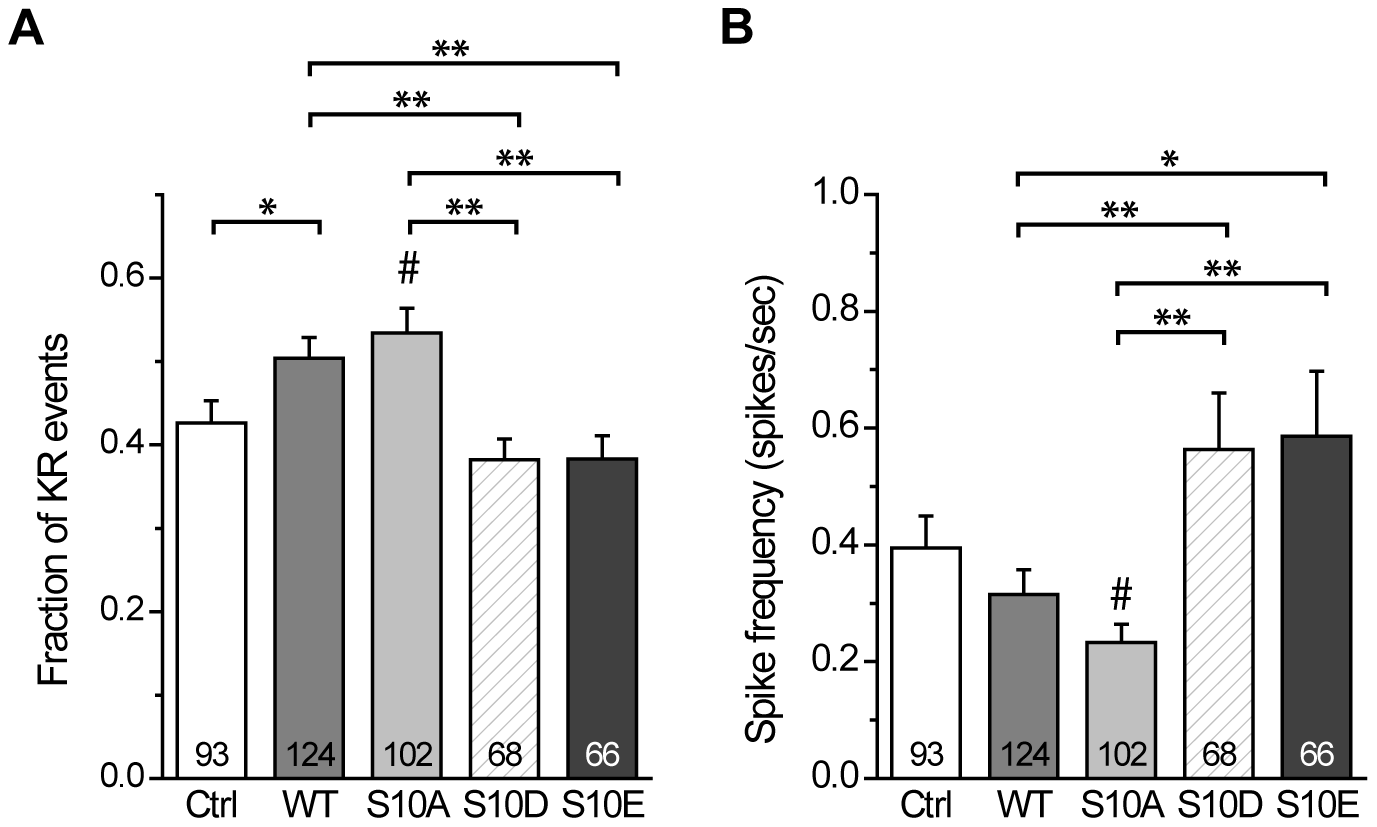
Fraction of KR events and spike frequency in cells overexpressing CSP and its phosphomutants. **A**, Fraction of KR events for the different groups. This fraction was calculated using the cellular mean method as the ratio of event numbers (i.e, number of 2–3.5 pA events divided by number of all events). Total 513–804 KR events from those cells. **B**, Spike frequencies for each group calculated using the cellular mean method from the start of KCl treatment to the end of recording (25 sec). Signals with peak amplitudes ≥3.5 pA were considered as spikes. Numbers in bars indicate the number of cells analyzed. Total 593–977 spikes from those cells.

To quantify the effect of CSPα phosphomutation on the occurrence of KR events, we compared the fraction of KR events (X_KR_) across all groups (Fig. 5A). We found that the X_KR_ for WT was significantly higher than that for Ctrl (*p*<0.05). Moreover, it was significantly higher than that for S10D (*p*<0.01) and for S10E (*p*<0.01). The X_KR_ for WT was comparable to that for S10A, but the X_KR_ for S10A was significantly higher than that for S10D (*p*<0.01), for S10E (*p*<0.01), and for Ctrl (*p*<0.05). These results suggest that CSPα phosphodeficiency increases the occurrence of KR events, and thus may play a role in regulating KR exocytosis.

### CSPα phosphodeficient mutation decreases the frequency of full fusion

Since S10A regulates the fraction of KR events, we next examined if it affects the frequency of full-fusion events. We assessed the amperometric recordings for signals that had peak amplitudes ≥3.5 pA in the same dataset used for the analysis of KR events. We calculated spike frequency using the cellular mean method (Fig. 5B). These frequencies across Ctrl and WT were comparable. In contrast, spike frequency for WT was significantly less than that for S10 D (*p*<0.01) and S10E (*p*<0.05), and spike frequency for S10A was significantly less than that for S10 D (*p*<0.01), S10E (*p*<0.01), and Ctrl (*p*<0.05), suggesting that CSPα phosphodeficiency decreases the frequency of full-fusion events. However, CSPα phosphomutation did not affect other spike characteristics, including spike height, half-width, rise time, decay time, and whole area (Table S1), suggesting that this phosphorylation site may not affect the filling of LDCVs or the post-dilation events after fusion pore opening.

### CSPα phosphomutation modulates fusion pore dynamics

Our amperometric recordings indicate that S10A decreases the secretion rate, increases the fraction of KR events, and decreases the frequency of full-fusion events compared to Ctrl. In addition, S10E prolongs the opening of the initial fusion pore that leads to dilation compared to Ctrl or WT. With these results we can determine the fusion pore kinetics that are regulated by CSPα phosphomutation. The previously proposed kinetic model for fusion pores [3], [6], [8], [36], [37], [38], [39] defined that the transition states of fusion pores as closed state (C), open state (O), and dilation state (D). The rate constant of the step from C to O is defined as *k_o_*, for the step from O to C as *k_c_*, and for the step from O to D as *k_d_*.
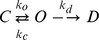



The rate constants *k_c_* and *k_d_* can be calculated using the following equations derived from single-channel kinetics [3], [6], [30], [38], [44], [45], [46], [47]:
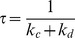


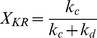



We took the duration t_1_ as the measure for the open time of KR events (τ) (Figure S4-A). Both S10D and S10E had the longer KR open time compared to WT or S10A. In addition, the KR open time in S10E was significantly longer compared to Ctrl (Figure S4-A). Together with the fraction of KR events (X_KR_) (Fig. 5A), *k_c_* and *k_d_* can be addressed by these two equations.

Using these values from Fig. 5A and Figure S4-A, we calculated *k_c_* and *k_d_* for each group (Figure S4-B and C, respectively). With CSPα phosphomimetic mutation (i.e., S10D and S10E), *k_c_* decreased (Figure S4-B), suggesting that this phosphomutation prevents fusion pores from leaving the open state and entering the closed state. Thus, CSPα-Ser^10^ phosphomimetic mutation stabilizes an open fusion pore (Figure S4-F). In contrast, both WT and S10A increased *k_c_* (Figure S4-B) but decreased *k_d_* (Figure S4-C), leading to the KR open time remained unchanged (Figure S4-A), but the fraction of KR events was significantly increased (Fig. 5A). Similar conclusion was obtained when the rate constants were resolved from the mean PSF lifetime (Figure S4-D for *k_c_*; Figure S4-E for *k_d_*). Given that the CSPα phosphomutants regulate the rate constants for the kinetic steps, CSPα-Ser^10^ phosphomutation may play an important role in modulating fusion pore dynamics.

## Discussion

In this study, we show that CSPα phosphodeficient mutation decreases the secretion rate, increases the fraction of KR events, and decreases the frequency of full fusion compared to the control. In contrast, CSPα phosphomimetic mutation prolongs the lifetime of the PSF compared to the control. These physiological effects cannot be attributed to changes in the levels of Syt I and SNARE proteins, or to aberrant subcellular targeting of CSP. Our kinetic analysis suggests that CSPα phosphomimetic mutation promotes fusion pore openings mainly by inhibiting open pores from closing. Thus, the phosphomimetic mutation of CSPα-Ser^10^ may regulate exocytosis by modulating fusion pore kinetics. The residue Ser^10^ of CSPα may serve as a converged residue that allows diverse cellular signals to regulate the function of CSPα in regulated exocytosis.

Several mechanisms could underlie the regulation of fusion pore kinetics by CSPα-Ser^10^ phosphomutation. Studies have shown that deleting the chaperone CSPα reduces the expression level of SN25 due to degradation of the misfolded SN25 [13], [14]. However, here we exclude this possibility since CSPα-Ser^10^ phosphomimetic mutation has no apparent effect on the expression levels of the essential exocytotic proteins, including Syt I and SNARE proteins (Figure S1-C). Our results suggest that the regulation of fusion pore kinetics by CSPα phosphomimetic mutation cannot be attributed to the altered chaperone function of CSPα, consistent with previous results showing that CSPα-Ser^10^ phosphorylation does not affect formation of the CSPα-Hsc70 chaperone complex [16], [26].

It is also unlikely that the regulation of fusion pore kinetics by CSPα phosphomimetic mutation is due to changes in intracellular Ca^2+^ levels. Studies of the fusion pore kinetics [6] indicate that the rate constant of fusion pore closure (*k_c_*) does not significantly depend on the intracellular Ca^2+^ levels. However, the rate constant for fusion pore dilation (*k_d_*) does increase with higher intracellular Ca^2+^ levels following a sigmoidal Ca^2+^ dependence, indicating that Ca^2+^'s action on fusion pore dilation is a cooperative process [6]. Here, we find that CSPα phosphomimetic mutation reduces *k_c_* but just slightly affecting *k_d_* (Figure S4-B, C, D, and E), suggesting that CSPα phosphomimetic mutation causes little change, if any, in intracellular Ca^2+^ levels in PC12 cells. These results are consistent with previous findings that CSPα can directly regulate exocytosis without altering Ca^2+^ entry, probably by influencing a step downstream of Ca^2+^ binding to the exocytotic machinery [16], [20], [21], [25], [48].

One plausible mechanism for the regulation of fusion pore kinetics by CSPα-Ser^10^ phosphomimetic mutation stems from this mutation enhancing the interaction between CSPα and Syx I (Text S1 and Figure S5). Syx I has residues in its transmembrane domain that face the lumen of the open fusion pore [49]. Mutations that selectively weaken the Syt I-Syx I binding do regulate *k_c_* [5]. Thus, it is likely that CSPα phosphomimetic mutation inhibits *k_c_* by increasing its binding to Syx I. How these changes in biochemical interactions lead to modulation of fusion pore dynamics requires further investigation. In addition, whether CSPα-Ser^10^ phosphorylation alters its interaction with other SNARE proteins (i.e., SN25 and Syb) to regulate fusion pore kinetics requires further investigation.

Another plausible mechanism by which CSPα phosphomimetic mutation regulates fusion pore kinetics comes from the finding that CSPα-Syt binding alters after CSPα-Ser^10^ phosphorylation [15], [16]. Many studies have shown that fusion pore kinetics are regulated by various Syt isoforms [2], [3], [4], [5], [6], [8], [39], [50]. For example, overexpression of Syt I or Syt IX stabilizes the initial fusion pore prior to dilation [3], [8]. Notably, phosphorylation of CSPα reduces its interaction not only with Syt I [15], but also with Syt IX [16], the other major Syt isoform in PC12 cells [8], [39], [51]. Hence, CSPα phosphorylation may globally affect the role of this Ca^2+^ sensor protein family in regulating fusion pore kinetics.

In addition to conventional SNARE proteins and Syt isoforms, CSPα has been shown to interact with dynamin I, a protein essential for vesicle fission, suggesting that CSPα may regulate synaptic vesicle endocytosis and facilitate exo- and endocytotic coupling [29]. Consistent with this finding, our results suggest that phosphorylation of CSPα may regulate fusion pore dynamics. How CSPα phosphorylation may affect its interaction with dynamin I requires for further investigation. Moreover, phosphorylation of CSPα-Ser^10^ has been found to trigger the binding with 14-3-3 protein, a protein implicated in a range of roles including chaperone function, neurodegeneration, and exocytosis [52]. Thus, through changing the phosphorylated state by cellular signalings, CSPα may have a great capacity to interact with the downstream target proteins and modulate multiple neuronal functions.

Previous studies have produced conflicting results on the role of CSPα in Ca^2+^-dependent exocytosis, especially with experiments of CSPα overexpression [13], [14], [20], [21], [22], [23], [24], [25], [48]. These discrepancies may arise from different methods for data analysis of amperometric spikes. In chromaffin cells, these spikes can have the altered shapes [26] or areas [21], [28] when wild-type or phosphodeficient CSPα is overexpressed. However, spike characteristics sometimes depend strongly on the cells producing the spikes [53], [54]. Since the pooled data of spike characteristics may lead to over-representation of a few dominant cells within one group, the double-mean method (i.e., the cellular mean method) should be used to reduce this bias. Furthermore, the discrepancies in CSPα overexpression experiments may also result from different cell types, culture conditions, or recording conditions, with different phosphorylation levels of endogenous CSPα [26], [48]. These results strongly suggest that it is important to examine the effects of both phosphomimetic and phosphodeficient mutants and compare those effects with WT in the same type of cells. In our study, we found that the effects of WT were similar to those of S10A but were unlike those of S10D or S10E, consistent with that most CSPα proteins in WT-overexpressed PC12 cells are hypophosphorylated (Figure S5). Therefore, the phosphorylation levels of WT-CSPα may switch its function in regulating Ca^2+^-dependent exocytosis.

Some particular types of cells confer a high PKA or PKB activity, such as the developing neurons [55], [56] and active endocrine cells [57], [58]. Since CSPα-Ser^10^ is phosphorylated by both PKA and PKB, CSPα phosphorylation is relatively important for promoting neurotransmitter and hormone release in these types of cells rather than PC12 cells in this study. Moreover, these protein kinases are activated by the second messengers that have shown to confer distinct spatiotemporal distributions in the cells [55], [56]. Through dynamically changing its phosphorylated state *in situ*, CSPα may have a great capacity to regulate the rate of regulated exocytosis. Hence, CSPα-Ser^10^ phosphorylation may provide a way for diverse cellular signalings to converge and regulate the kinetics of exocytosis. Given that loss of CSPα function associates with many neurological diseases [59], [60], understanding the precise role of CSPα phosphorylation in regulated exocytosis is critical to unveil the mechanisms that underlie pathological conditions.

## Materials and Methods

### Molecular biology

The DNA encoding wild-type CSPα1 (WT) was kindly provided by Dr. Cameron Gundersen (UCLA). It was subcloned into pIRES2EGFP (Clontech #6029-1) by polymerase chain reaction (PCR) with 30 cycles of 94°C for 1 min, 63°C for 1 min, and 72°C for 2 min. The primers annealing the upstream *Xho*I and downstream *Sal*I sites were 5′-CTC ATA GTT ACT AAC TCG AGA TGG CTG ACC-3′ and 5′-CAC AGC CTC TCG TCG ACT TAG TTG AAC-3′, respectively. The PCR product was digested by *Xho*I (Takara #1094A) and *Sal*I (Takara #1080A), and then inserted into pIRES2EGFP with ligation kit (Takara #6022). The resultant plasmid (pIRES2EGFP-CSPα1) was transformed into Top10 competent cells (Invitrogen #C4040-10) and amplified in the Luria broth (LB) agar plates containing kanamycin (50 µg/mL, Sigma #K4000). The DNA plasmid was purified (Qiagen #12181) and dissolved in Tris buffer (10 mM, pH 7.6).

The DNA plasmids encoding CSPα1 phosphomutants (S10A, S10D and S10E) were prepared by modified sequential PCR [61] with pIRES2EGFP-CSPα1 as the template. The phosphodeficient mutant (S10A) was prepared by replacing serine with alanine, and the phosphomimetic mutants (S10D and S10E) were prepared by replacing serine with negatively-charged amino acids, aspartate and glutamate, respectively. Two pairs of primers were used for modified sequential PCR. One pair of primers annealing the upstream *Bgl*II and downstream *Sac*II sites were 5′-GGG ACT TTC CTA CTT GGC AGT ACA TCT ACG-3′ and 5′-CTT ATT CCA AGC GGC TTC GGC CAG TAA C-3′, respectively. The other pair of primers (forward and reverse) were designed to introduce the point mutations at Ser^10^ of CSPα1. To introduce the S10A point mutation, the forward and reverse primers were 5′-CAG CGC TCA CTC GCT ACT TCC GGG GAA TC-3′ and 5′-GAT TCC CCG GAA GTA GCG AGT GAG CGC TG-3′, respectively. (Note, here and below the underlined deoxynucleotides were complementary sequences to the specific site). To introduce the S10D point mutation, the forward and reverse primers were 5′-CAG CGC TCA CTC GAT ACT TCC GGG GAA TC-3′ and 5′-GAT TCC CCG GAA GTA TCG AGT GAG CGC TG-3′, respectively. To introduce the S10E point mutation, the forward and reverse primers were 5′-CAG CGC TCA CTC GAA ACT TCC GGG GAA TC-3′ and 5′-GAT TCC CCG GAA GTT TCG AGT GAG CGC TG-3′, respectively.

Three runs of PCR were included in modified sequential PCR. For the first run of PCR, one tube held the upstream primers, reverse primers containing mutation sites, template DNA, DNA polymerase (LA *Taq*, Takara #RR002A), and dNTPs, while the other tube held the downstream primers, forward primers containing mutation sites, template DNA, DNA polymerase, and dNTPs. The DNA fragments in both tubes were amplified by 30 cycles of 94°C for 1 min, 60°C for 2 min, and 72°C for 2 min. The first-run-PCR products were gel-extracted, purified (Qiagen #28704), and then combined for the second run of PCR using the same conditions except with only 10 cycles. The second-run-PCR product was added to the upstream primers, downstream primers, dNTPs, and DNA polymerase for the third run of PCR with 25 cycles of 94°C for 1 min, 52°C for 1 min, and 72°C for 2 min. The final PCR product was gel-extracted, purified, digested by *Bgl*II (Takara #1021A) and *Sac*II (Takara #1079A), and ligated into pIRES2EGFP.

The DNA plasmids encoding CSPα1 phosphomutants (S10A, S10D and S10E) were confirmed by automated sequencing, amplified, and purified for transfection.

### Cell culture and transient transfection

PC12 cells [3], [62] were cultured in 10 cm dishes (Corning #430167) in a medium containing Dulbecco's modified Eagle's medium (DMEM, Sigma #D5648), 3.7 g/ml NaHCO_3_, 5% Equine serum (HyClone #SH30074.03) and 5% bovine calf serum (HyClone #SH30072.03) at 37°C in a 10% CO_2_ humidified incubator (MCO-5AC, Sanyo). The medium was renewed every other day, and the cells were passed into new dishes upon full confluence (∼5 days).

For transient transfection, cells were washed and suspended with Hank's solution, consisting of Hanks' balanced salts (Sigma #H4891), 0.35 g/ml NaHCO_3_, and 1 mM EGTA, pH 7.2. The cells were then centrifuged at 2,000 *g* for 3 min. The supernatant was discarded, and the cell pellet was resuspended in 500 µL of Cytomix (120 mM KCl, 0.15 mM CaCl_2_, 10 mM KH_2_PO4, 2.5 mM HEPES, 2 mM EGTA, and 5 mM MgCl_2_, pH 7.6). The cell suspension was further mixed with 50 µg of DNA plasmid and transferred into a 4 mm cuvette (BTX ECM830, Harvard Apparatus #45-0126). Transfection was performed by electroporation with a pulse duration of 5 msec and a voltage of 230 V (BTX ECM830, square-pulse electroporator, Harvard Apparatus #04-001-1A). Transfected cells were immediately transferred into culture medium containing 10% fetal bovine serum (Biological Industries #04-004-1A). The medium was renewed on the following day. The cells expressing genes of interest were identified by green fluorescence at 2–5 days after transfection.

### Amperometry and data analysis

PC12 cells [3], [62] were harvested at 48 hr post transfection using a 22 gauge needle (Terumo #SS-10L2238), re-plated onto 3.5 cm collagen I- and PDL-coated dishes at a density of 2×10^5^ per dish, and cultured for 4 hr. The cells were then incubated in culture medium containing 1.5 mM norepinephrine (NE) (Sigma #N5785) and 0.5 mM ascorbate (Sigma #A5960) for 16 hr. Culture medium was replaced by fresh medium at least 1 hr prior to amperometric recordings. During recordings, cells were incubated in a bathing solution of 150 mM NaCl, 4.2 mM KCl, 1 mM NaH_2_PO_4_, 0.7 mM MgCl_2_, 2 mM CaCl_2_ and 10 mM HEPES, pH 7.4 [3].

Amperometric recordings were conducted at 22°C using a 5 µm carbon fiber (CFE-1, ALA Scientific Instruments) connected to a VA-10× amplifier (ALA Scientific Instruments) at a polarization of 650 mV. The freshly-cut carbon fiber was gently attached to the cell, and Ca^2+^-dependent exocytosis was induced by puffing a high-K^+^ solution (105 mM KCl instead of NaCl in the bathing solution) from a 2 µm micropipette. For each trial of recordings, solutions were ejected for 20 sec with pressure (10–20 p.s.i.) gated by a Picospritzer (General Valve Corp.). Each cell was recorded for five consecutive trials. NE released from the cell was detected by the potentiated carbon fiber that was connected to the amplifier and a computer running pClamp 10 software (Axon Instruments, Molecular Devices Corp.). The signal was digitized at 4 kHz and low-pass-filtered by eight-pole Bessel at 1 kHz [3].

Amperometry data were analyzed using a computer program written in the previous studies [3], [6], [35] to extract PSF information according to the criteria of Chow and von Rüden [63]. Events with cut-off peak amplitudes above 2 pA (∼7×RMS noise) were collected. Large spikes (peak amplitude ≥13 pA) were used for analysis of PSF. The PSF lifetime was measured from onset (the current rising to 1×RMS noise above the baseline current) to end point (the intersection between the baseline and the line going through the rise phase from 35 to 60% of the peak) [63] (Fig. 2A). The PSF area was integrated in this time interval (Fig. 2A, shaded area). The PSF mean amplitude was calculated from the area divided by the lifetime. The PSF with lifetimes under 0.75 msec (3×sampling interval) were excluded, yielding ∼24% spikes without PSF detected. The mean PSF duration (τ) (Fig. 2B) was determined by fitting the distribution of PSF lifetimes to an exponential function using the computer program Origin 8 (OriginLab Corp.).

Methods of analysis were developed previously [6] to distinguish the KR events from those full-fusion spikes that are distorted by diffusion [64]. Generally, once an event (with peak amplitude ≥2 pA) was identified by the computer program, the onset was taken as that used for PSF as mentioned above. The end of a putative KR event was taken as the time when the signal passed below the average value of the points between the two preliminary time boundaries defined by the 50% peak amplitude. The duration was referred to as t_1_ and illustrated in the traces in Fig. 3B–C. The duration t_2_ was measured from the onset to the end point, taken as the time when signal returned to within 1×RMS noise of the baseline current (Fig. 3B–C). The ratios of these two times (t_1_/t_2_) provided an index of event shape that was used to evaluate the rectangularity of KR events (Fig. 3C–D). The mean amplitude of KR events (Fig. 4A–B) was calculated from the integrated area divided by the duration t_1_ for comparison with PSF mean amplitude. We chose the duration t_1_ as the measure of KR open time because this duration is very sensitive to event shape and can distinguish the KR events from the events occurring at remote distance. During recording, if the carbon fiber electrode was not attached to the cell membrane very well, the events occurring at remote distance of the electrode would be detected and those events were distorted due to diffusion, resulting in small peak amplitude and long duration. In this scenario, the duration t_1_ usually became much longer (> several tens of msec), in contrast to the relatively brief duration t_1_ in the square-like events (∼7 msec, Figure S4-A).

The features of spikes (≥3.5 pA) were also analyzed by the written computer program (Fig. 2A). Spike height was the peak amplitude rising from the 1×RMS noise above the baseline. Half-width was the time interval between the two points at the 50% peak amplitude. The rise time was taken as the time for the spike to rise from 35 to 90% of peak amplitude. The decay time was taken as the time to fall from the peak to within 1×RMS noise of the baseline. The whole area was taken from the data integrated in the time interval between the onset (the signal rising to 1×RMS noise above the baseline current) and end point (the signal returned to within 1×RMS noise of the baseline current).

### Statistics

All data were reported as mean ± S.E.M. (Origin 8, OriginLab Corp.). The double-mean method (i.e., the cellular mean method) was used to calculate the average of cellular means from individual cells to eliminate variations across cells [6], [53]. Differences between the cellular means of two groups were evaluated for statistical significance using the two-tailed Student's unpaired *t*-test for the parametric method, and the Mann-Whitney method for the nonparametric method. Differences between the cellular means of larger groups (≥3 groups) were evaluated with the One-way ANOVA followed by the *post*-*hoc* Student-Newman-Keuls test for the parametric method, and the Kruskal-Wallis method followed by the *post*-*hoc* Dunn test for the nonparametric method. Asterisks indicated significance in the following manner: *, *p*<0.05; **, *p*<0.01; ***, *p*<0.001 among two groups; #, *p*<0.05 in comparison with the control group (InStat 3, GraphPad).

## Supporting Information

Figure S1 **The expression levels of CSP and other essential exocytotic proteins after transfection.** **A**, Relative mRNA levels of CSP isoforms in the Ctrl cells (normalized to β-actin, n = 3). Inset, CSPα splicing variants analyzed by RT-qPCR. CSPα1 corresponds to the 196 bp PCR product and CSPα2 to the 268 bp fragment. Left lane shows DNA length marker. **B**, Relative mRNA levels of CSP isoforms in cells transfected with WT-CSPα or its phosphomutants (normalized to β-actin and Ctrl, n = 3). *p<0.05, two-tailed Student's unpaired *t*-test. **C**, Protein levels of CSP, Syt I, Syx I, SN25, and Syb relative to α-tubulin in cells transfected with control vector pIRES2EGFP (Ctrl), WT-CSPα, or its phosphomutants. **D**, Immunofluorescent images show the distribution of CSP (green) after KCl depolarization. EGFP (white) indicates cells with successful transfection in different groups. BF, bright field. Scale bars, 5 µm.
(PDF)

Figure S2 **The zoomed traces of amperometric recordings in cells overexpressing the CSPα phosphomimetic mutant (S10E).** Three continuous traces (**A**–**C**) were obtained from different cells. Insets in A, the zoomed windows to show the shape for the events with different peak amplitudes (a–c). Event “a” with peak amplitude of 2.23 pA; Event “b” with peak amplitude of 3.54 pA; Event “c” with peak amplitude of 11.89 pA. X-axis, current (pA); Y-axis, recording time (msec).
(PDF)

Figure S3 **Histograms of spike mean amplitude in cells overexpressing CSP and its phosphomutants.** Histograms were constructed by spike “*mean*” amplitudes for all groups. Signals with peak amplitudes ≥3.5 pA were considered as spikes. Total 593–977 spikes from 66–124 cells.
(PDF)

Figure S4 **Kinetics of the fusion pore modulated by CSPα phosphomutation.** **A**, The open time of KR events in cells overexpressing CSP and its phosphomutants. Total 513–804 KR events from 66–124 cells out of the same datasets in Fig. 5. **B**, The rate constant pushing the fusion pore toward closure (k_c_) that was derived from the KR open time (A) and the KR fraction (Fig. 5A). **C**, The rate constant pushing the fusion pore toward dilation (k_d_) that was derived from the KR open time (A) and the KR fraction (Fig. 5A). **D**, The rate constant pushing the fusion pore toward closure (k_c_) that was derived from the PSF duration (Fig. 2C) and the KR fraction (Fig. 5A). **E**, The rate constant pushing the fusion pore toward dilation (k_d_) that was derived from the PSF duration (Fig. 2C) and the KR fraction (Fig. 5A). **F**, Kinetic model of pore opening, closing, and dilation. CSPα phosphomimetic mutation inhibits an open fusion pore from closing (the kinetic step shown in red).
(PDF)

Figure S5 **The binding with syntaxin I is increased in the CSP phosphomimetic mutants.** **A**, Phosphorylation levels in cells overexpressing CSP and its phosphomutants. PC12 cells transfected with WT, S10A, S10D, or S10E were treated with a high-K^+^ solution for 15 min, and cell lysates were prepared. CSP protein was immunoprecipitated (IP), and phosphorylation was determined by immunoblotting (IB) with anti-phospho-PKA substrate antibody. **B**, Phosphorylation of CSP-Ser^10^ modulates its interaction with syntaxin I (Syx I). Transfected PC12 cells were treated with a high-K^+^ solution for 15 min, and cell lysates were prepared. CSP protein was immunoprecipitated, and Syx I was determined by immunoblotting with anti-Syx I antibody. **C**, Phosphorylation of overexpressed CSP-WT is comparable with endogenous CSP under the resting and high-K^+^-stimulated conditions. PC12 cells transfected with the control vector pIRES2EGFP (Vector) or CSP-WT were treated without or with a high-K^+^ solution (KCl) for 15 min, and cell lysates were prepared. CSP protein was immunoprecipitated (IP), and phosphorylation was determined by immunoblotting (IB) with anti-phospho-PKA substrate antibody. Input control shows the levels of overexpression compared to the endogenous CSP.
(PDF)

Table S1 **Characteristics of spikes in cells overexpressing CSP and its phosphomutants.**
(PDF)

Text S1 **Methods: Reverse-transcriptase quantitative polymerase chain reaction (RT-qPCR), Western blot analysis, Immunofluorescence staining, and Immunoprecipitation.**
(PDF)
