# Supplementary figures and images for: Phosphomimetic Mutation of Cysteine String Protein-α Increases the Rate of Regulated Exocytosis by Modulating Fusion Pore Dynamics in PC12 Cells
Source: PLoS One. 2014 Jun 23;9(6):e99180. doi: 10.1371/journal.pone.0099180 (PMC4067274; doi:10.1371/journal.pone.0099180)

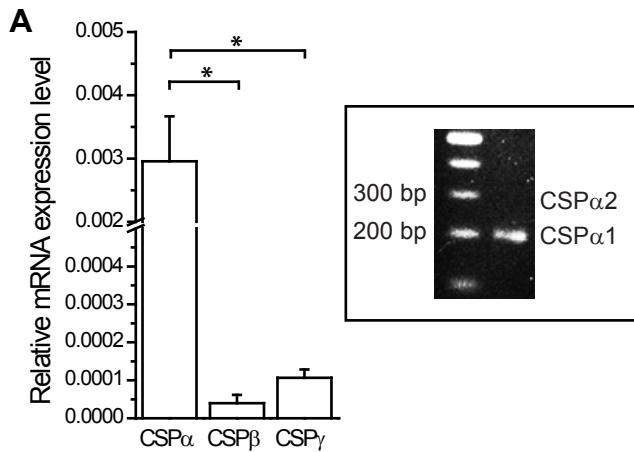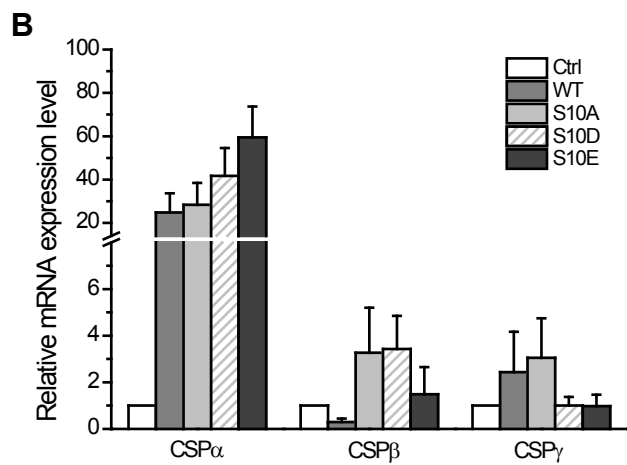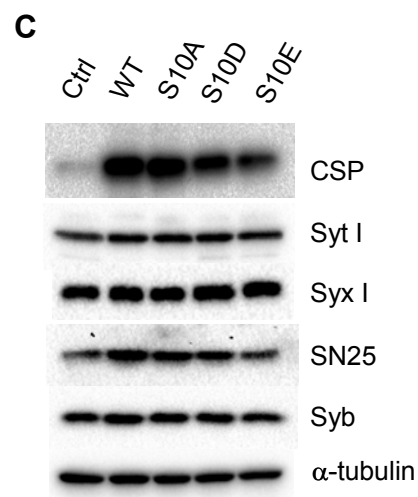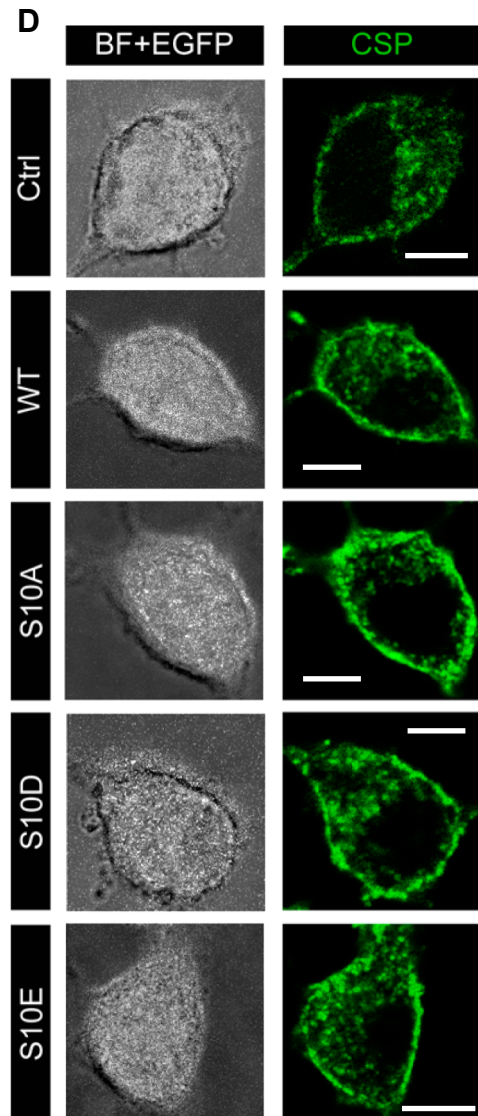

**Figure S1**

Supplement: Figure S1 — The expression levels of CSP and other essential exocytotic proteins after transfection. A, Relative mRNA levels of CSP isoforms in the Ctrl cells (normalized to β-actin, n = 3). Inset, CSPα splicing variants analyzed by RT-qPCR. CSPα1 corresponds to the 196 bp PCR product and CSPα2 to the 268 bp fragment. Left lane shows DNA length marker. B, Relative mRNA levels of CSP isoforms in cells transfected with WT-CSPα or its phosphomutants (normalized to β-actin and Ctrl, n = 3). *p<0.05, two-tailed Student's unpaired t-test. C, Protein levels of CSP, Syt I, Syx I, SN25, and Syb relative to α-tubulin in cells transfected with control vector pIRES2EGFP (Ctrl), WT-CSPα, or its phosphomutants. D, Immunofluorescent images show the distribution of CSP (green) after KCl depolarization. EGFP (white) indicates cells with successful transfection in different groups. BF, bright field. Scale bars, 5 µm. (PDF) [file pone.0099180.s001.pdf]

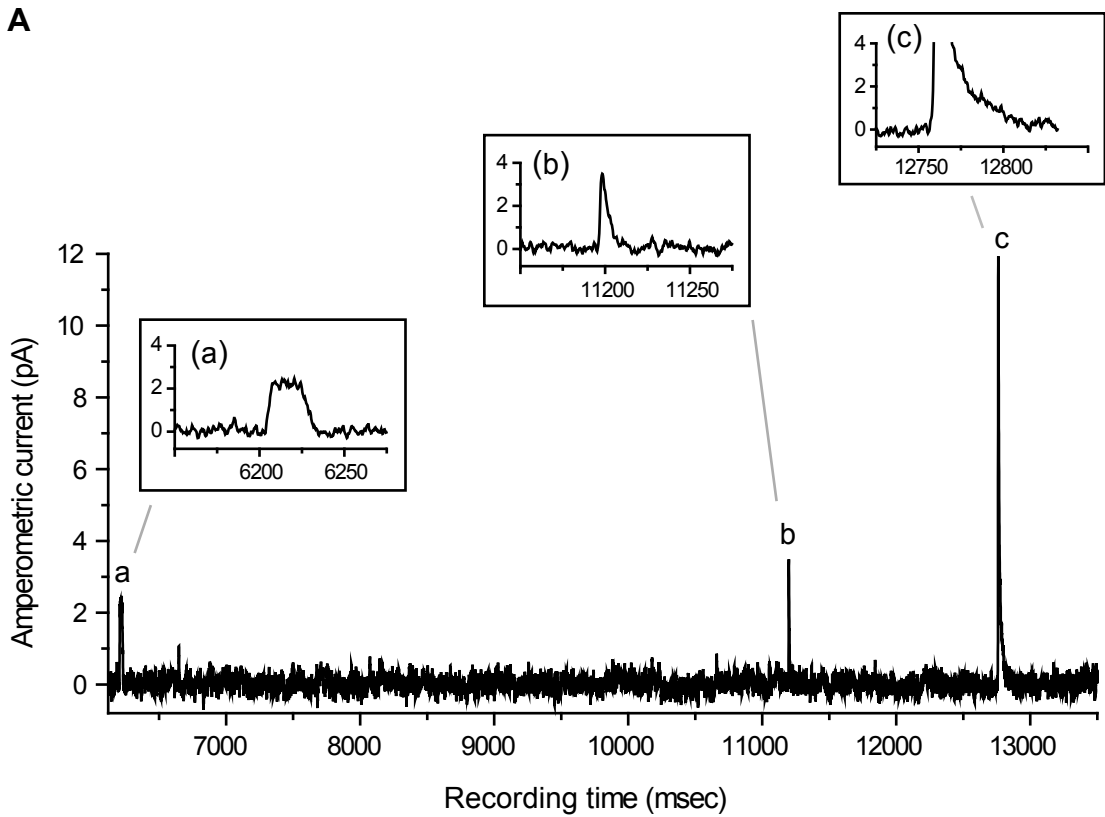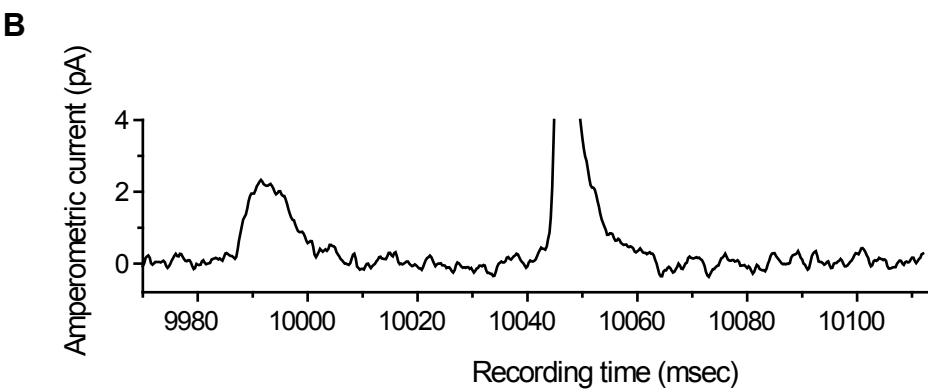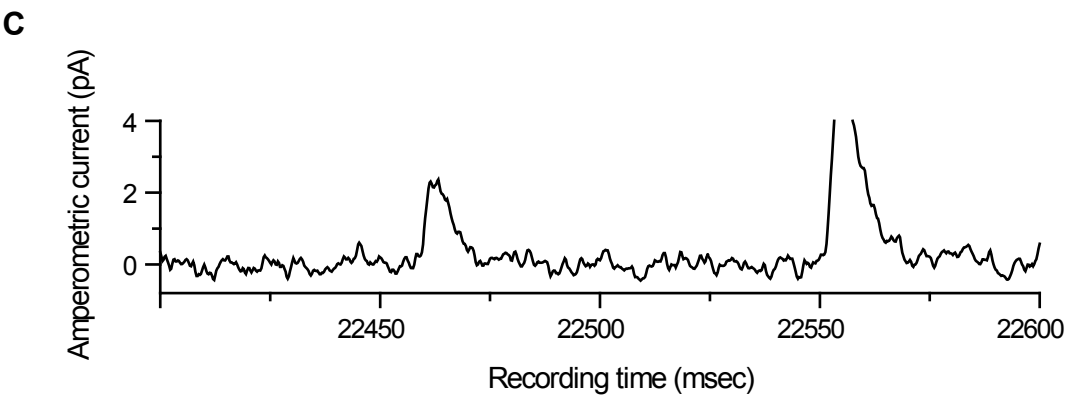

**Figure S2**

Supplement: Figure S2 — The zoomed traces of amperometric recordings in cells overexpressing the CSPα phosphomimetic mutant (S10E). Three continuous traces (A–C) were obtained from different cells. Insets in A, the zoomed windows to show the shape for the events with different peak amplitudes (a–c). Event “a” with peak amplitude of 2.23 pA; Event “b” with peak amplitude of 3.54 pA; Event “c” with peak amplitude of 11.89 pA. X-axis, current (pA); Y-axis, recording time (msec). (PDF) [file pone.0099180.s002.pdf]

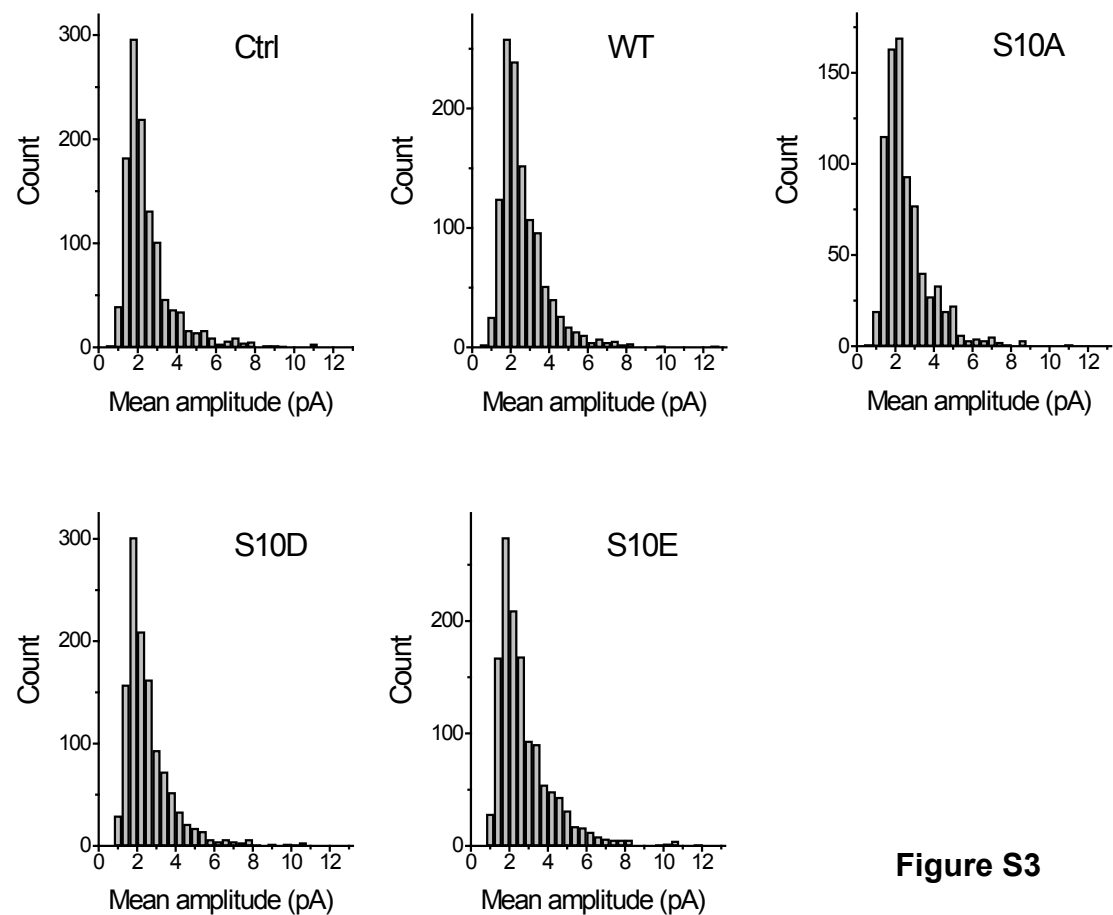

**Figure S3**

Supplement: Figure S3 — Histograms of spike mean amplitude in cells overexpressing CSP and its phosphomutants. Histograms were constructed by spike “mean” amplitudes for all groups. Signals with peak amplitudes ≥3.5 pA were considered as spikes. Total 593–977 spikes from 66–124 cells. (PDF) [file pone.0099180.s003.pdf]

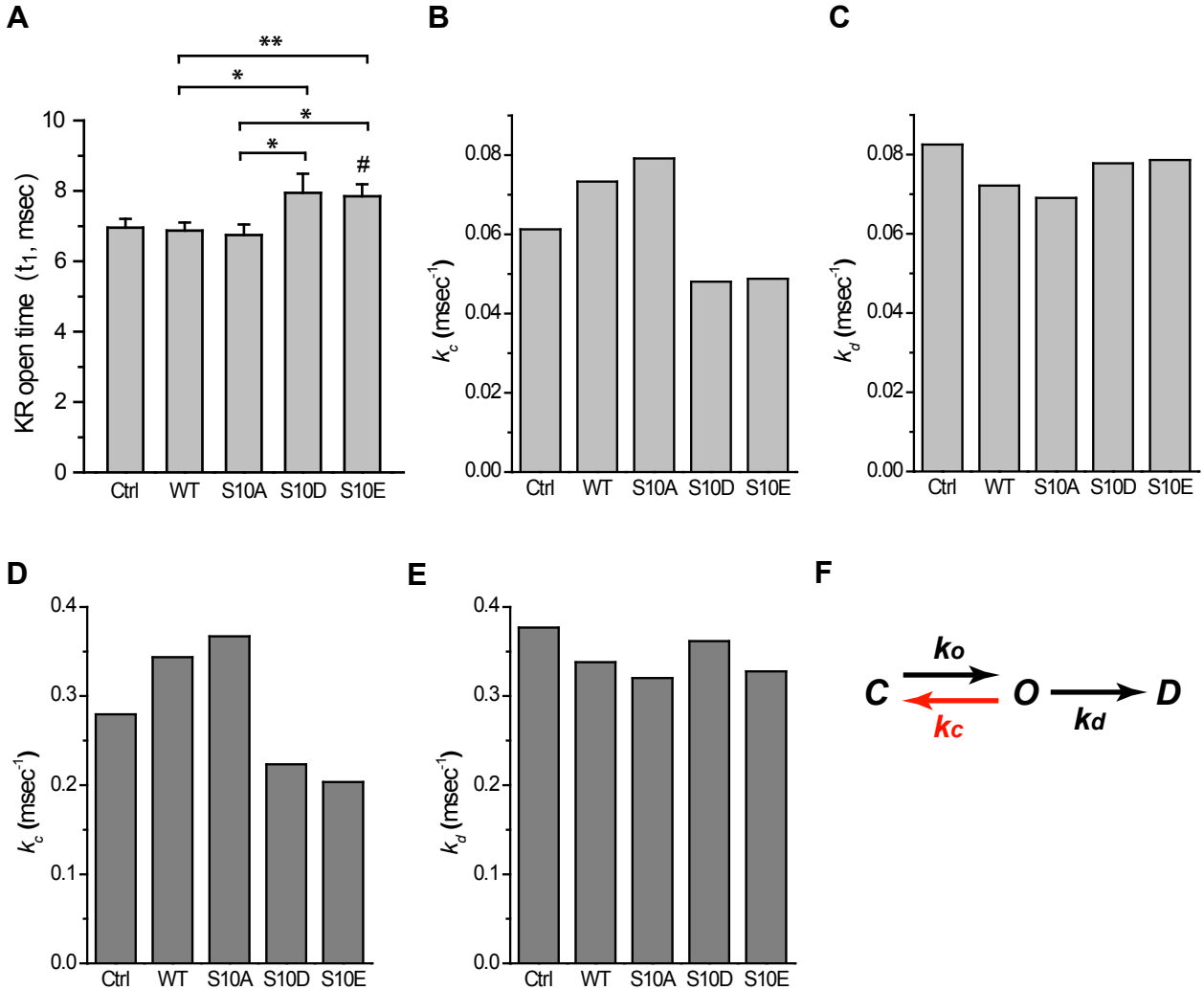

**Figure S4**

Supplement: Figure S4 — Kinetics of the fusion pore modulated by CSPα phosphomutation. A, The open time of KR events in cells overexpressing CSP and its phosphomutants. Total 513–804 KR events from 66–124 cells out of the same datasets in Fig. 5. B, The rate constant pushing the fusion pore toward closure (kc) that was derived from the KR open time (A) and the KR fraction (Fig. 5A). C, The rate constant pushing the fusion pore toward dilation (kd) that was derived from the KR open time (A) and the KR fraction (Fig. 5A). D, The rate constant pushing the fusion pore toward closure (kc) that was derived from the PSF duration (Fig. 2C) and the KR fraction (Fig. 5A). E, The rate constant pushing the fusion pore toward dilation (kd) that was derived from the PSF duration (Fig. 2C) and the KR fraction (Fig. 5A). F, Kinetic model of pore opening, closing, and dilation. CSPα phosphomimetic mutation inhibits an open fusion pore from closing (the kinetic step shown in red). (PDF) [file pone.0099180.s004.pdf]

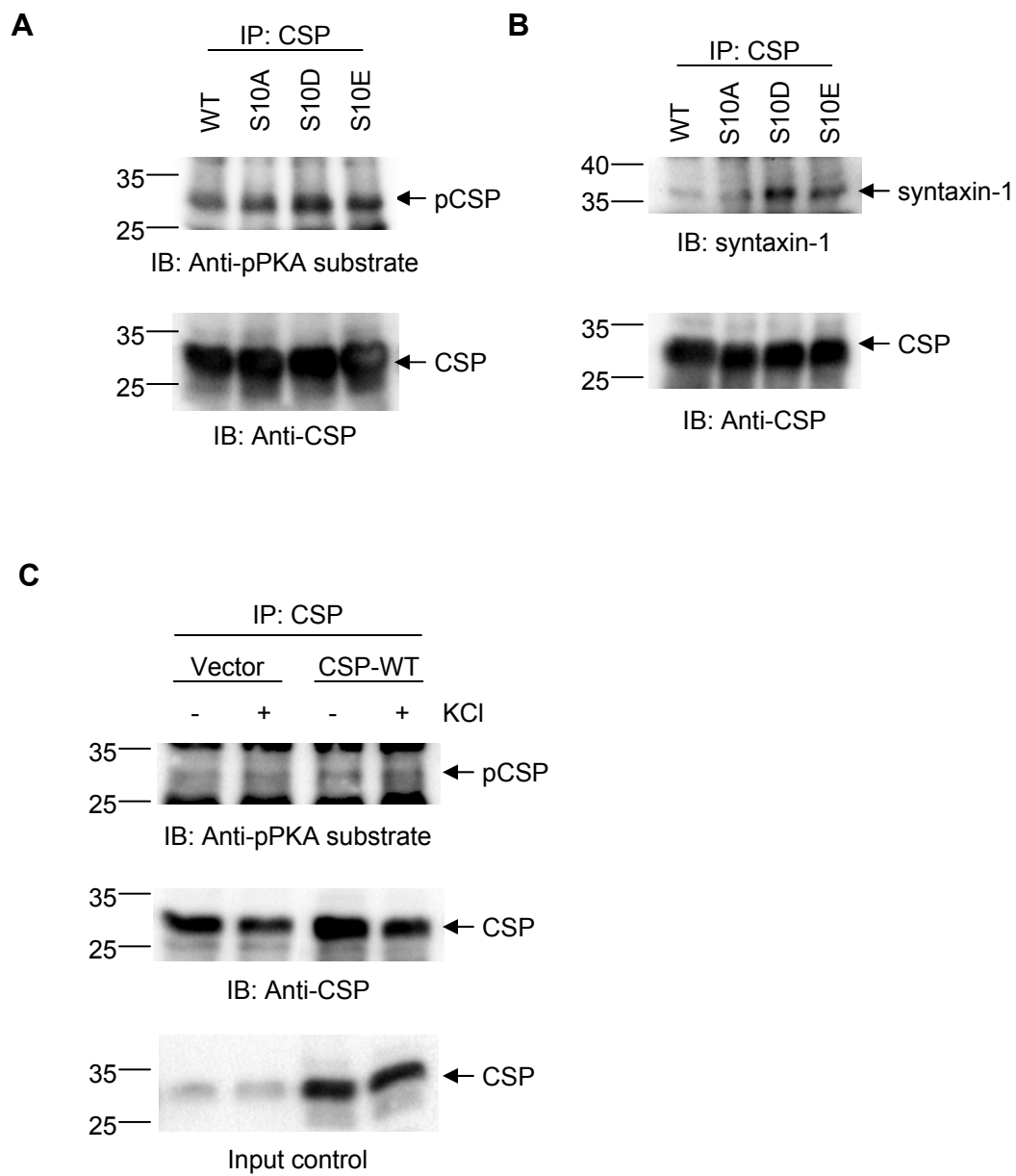

**Figure S5**

Supplement: Figure S5 — The binding with syntaxin I is increased in the CSP phosphomimetic mutants. A, Phosphorylation levels in cells overexpressing CSP and its phosphomutants. PC12 cells transfected with WT, S10A, S10D, or S10E were treated with a high-K+ solution for 15 min, and cell lysates were prepared. CSP protein was immunoprecipitated (IP), and phosphorylation was determined by immunoblotting (IB) with anti-phospho-PKA substrate antibody. B, Phosphorylation of CSP-Ser10 modulates its interaction with syntaxin I (Syx I). Transfected PC12 cells were treated with a high-K+ solution for 15 min, and cell lysates were prepared. CSP protein was immunoprecipitated, and Syx I was determined by immunoblotting with anti-Syx I antibody. C, Phosphorylation of overexpressed CSP-WT is comparable with endogenous CSP under the resting and high-K+-stimulated conditions. PC12 cells transfected with the control vector pIRES2EGFP (Vector) or CSP-WT were treated without or with a high-K+ solution (KCl) for 15 min, and cell lysates were prepared. CSP protein was immunoprecipitated (IP), and phosphorylation was determined by immunoblotting (IB) with anti-phospho-PKA substrate antibody. Input control shows the levels of overexpression compared to the endogenous CSP. (PDF) [file pone.0099180.s005.pdf]
