# Supplementary material for: Phosphomimetic Mutation of Cysteine String Protein-α Increases the Rate of Regulated Exocytosis by Modulating Fusion Pore Dynamics in PC12 Cells
Source: PLoS One. 2014 Jun 23;9(6):e99180. doi: 10.1371/journal.pone.0099180 (PMC4067274; doi:10.1371/journal.pone.0099180)
Supplement: Table S1 — Characteristics of spikes in cells overexpressing CSP and its phosphomutants. (PDF) [file pone.0099180.s006.pdf]

Table S1. Characteristics of spikes in cells overexpressing CSP and its phosphomutants.

	Spike height (pA)	*Half-width (msec)*	*35-90% Rise time (msec)*	*Decay time (msec)*	*Whole area (pA × msec)*	*# cells*
**Ctrl**	8.19 ± 0.48	5.65 ± 0.14	1.31 ± 0.04	19.57 ± 0.73	51.26 ± 2.78	87
**WT**	7.71 ± 0.25	5.93 ± 0.18	1.42 ± 0.05	19.61 ± 0.85	55.75 ± 2.97	107
**S10A**	7.23 ± 0.33	5.82 ± 0.16	1.33 ± 0.04	19.51 ± 1.14	48.21 ± 2.34	86
**S10D**	7.47 ± 0.33	5.58 ± 0.20	1.26 ± 0.05	19.92 ± 1.09	50.11 ± 2.56	65
**S10E**	7.58 ± 0.29	5.46 ± 0.20	1.22 ± 0.04	18.60 ± 0.80	48.54 ± 2.85	65

The characteristics of spikes (with peak amplitude ≥ 3.5 pA) were automatically measured by a computer program according to the criteria shown in Figure 3A. The cellular mean methods were used to calculate the spike height, half-width, rise time (35-90% to peak), decay time, and whole area. No significant differences were found between Ctrl and WT. In addition, no significant differences were found among WT, S10A, S10D, and S10E. Total 593-977 spikes from 66-124 cells.
