# Supplementary material for: Phosphomimetic Mutation of Cysteine String Protein-α Increases the Rate of Regulated Exocytosis by Modulating Fusion Pore Dynamics in PC12 Cells
Source: PLoS One. 2014 Jun 23;9(6):e99180. doi: 10.1371/journal.pone.0099180 (PMC4067274; doi:10.1371/journal.pone.0099180)
Supplement: Text S1 — Methods: Reverse-transcriptase quantitative polymerase chain reaction (RT-qPCR), Western blot analysis, Immunofluorescence staining, and Immunoprecipitation. (PDF) [file pone.0099180.s007.pdf]

Methods

Reverse-transcriptase quantitative polymerase chain reaction (RT-qPCR)

PC12 cells [3,62] were harvested after 48 hr post transfection by centrifuging at 12,000 *g* at 4°C for 5 min. The supernatant was discarded, and the cell pellet was acutely frozen by liquid nitrogen. One microliter of TRIzol (Invitrogen #15596-018) was added to lyse the cell pellet while grinding it with a pestle. The lysate was incubated in TRIzol at room temperature for 5 min and then 200 μL chloroform (Sigma #C2432) was added. The sample was vigorously shaken for 15 sec and then incubated at room temperature for 3 min. The lysate was centrifuged at 12,000 *g* at 4°C for 15 min, allowing its separation into three phases. The uppermost aqueous phase containing RNA was transferred into a new tube with 500 μL isopropanol (Sigma #I9516). The sample was inverted 5 times, incubated at room temperature for 10 min, and then centrifuged at 12,000 *g* at 4°C for 10 min. The supernatant was discarded, and 1 mL of 75% ethanol was added. The sample was inverted once and centrifuged at 12,000 *g* at 4°C for 10 min. The supernatant was removed, and the RNA pellet was air-dried at room temperature. The pellet was dissolved in 20 μL of RNase-free water and incubated in a 60°C water bath for 15 min. The final RNA sample was stored at -80°C.

The RNA samples were reverse-transcribed into first-strand cDNAs by Omniscript Reverse Transcriptase Kit (Qiagen # 205110, Germany). The cDNA products were dissolved by adding KAPA SYBR FAST qPCR Master Mix (Kapa Biosystems # KK4600), and were supplemented with upstream and downstream primers to perform qPCR under conditions of 95°C for the first 3 min followed by 40 cycles each of 95°C for 3 sec, 60°C for 45 sec, and 72°C for 30 sec. The upstream and downstream primers, respectively, were 5'-TGC TCT GGC TCC TAG CAC CAT GAA GAT CAA-3' and 5'-AAA CGC AGC TCA GTA ACA GTC CGC CTA GAA-3' for β-actin, 5'-GCC AAG GCG CTG TTC GCT GT-3' and 5'-TCG GTG TGT CTG TAG CCT CCC T-3' for CSPα, 5'-GCT GGT GGG CAA AGA CCC TGT-3' and 5-TGG TGT CGA TGT CTT GGG CCG-3' for CSPβ, and 5'-AGC CTA CAG GAA ACT GGC CTT GC-3' and 5'-GCG AGC CGT GCC GGT CAT AG-3' for CSPγ. The upstream and downstream primers used to distinguish CSPα1 and CSPα2 mRNA were 5'-GCC AAG GCG CTG TTC GTC GT-3' and 5'-TCG GTG TGT CTG TAG CCT CCC T-3', respectively.

Fluorescence data from qPCR was collected during every cycle of extension, and analyzed by the qPCR machine (Qiagen Rotor-Gene Q) using supplemental software (Qiagen Rotor-Gene Series Software 1.7). The cycle of threshold (Ct), defined as the intersection on the threshold vs. amplification plot, was recorded for the internal control (β-actin) and the target genes (CSPα, CSPβ, and CSPγ). The value of Ct was analyzed using the method of ΔΔCt. Briefly, we first obtained ΔCt by subtracting the Ct of the internal control (β-actin) from the Ct of the target genes, and we then obtained ΔΔCt by subtracting the ΔCt of the Ctrl group (pIRES2EGFP) from the ΔCt of the other transfection groups. The relative expression levels of the target genes were calculated as $2^{(-\Delta\Delta Ct)}$ and were used to compare the transfection groups with the Ctrl group.

Western blot analysis

PC12 cells were extracted using the ice-cold RIPA lysis buffer [1% NP-40, 150 mM NaCl, 1 mM EDTA, 1 mM phenylmethylsulfonyl fluoride, 1 mM sodium orthovanadate, 1 mM sodium fluoride, and 50 mM HEPES, pH 7.4, supplemented with protease inhibitor cocktail (Sigma #P2714)]. Twenty microliters of cellular protein lysate were electrophoresed via standard 12-15% Laemmli SDS polyacrylamide gels and transferred to polyvinylidene fluoride membranes. Membranes were blocked for 1 hr in 5% non-fat milk in TBST (100 mM Tris-HCl, pH 7.5, 150 mM NaCl, and 0.1% Tween-20), and were then incubated with primary antibodies [rabbit anti-CSP (Chemicon #AB1576), 1:5000; mouse anti-Syt I (Synaptic Systems #105011), 1:10000; mouse anti-Syx I (Santa Cruz #sc-12736), 1:8000; mouse anti-SN25 (Synaptic Systems #111011), 1:10000; mouse anti-Syb (Synaptic Systems #104211), 1:40000; mouse anti-α-tubulin (Sigma #T5168), 1:200000] at 4°C overnight. Membranes were washed three times with TBST and then incubated with secondary antibody in 5% milk in TBST at room temperature for 1 hr. Membranes then were washed three times with TBST, and signals were visualized by enhanced chemiluminescence followed by autoradiography.

Immunofluorescence staining

For immunostaining, PC12 cells [3,62] at 60 hr post transfection were plated on coverslips previously coated with collagen I (5 μg/cm^2) (BD Biosciences #354246) and

poly-d-lysine (PDL) (5 μg/cm^2) (BD Biosciences #354210). After attaching to the coverslips, the cells were first treated with a high-K^+ solution (105 mM KCl, 5 mM NaCl, 1 mM NaH_2PO_4, 0.7 mM $MgCl_2$, 2 mM $CaCl_2$, 10 mM HEPES, pH 7.4 with KOH) for 5 min, further washed with phosphate buffered saline (PBS: 137 mM NaCl, 2.7 mM KCl, 4.3 mM Na_2HPO_4, 1.47 mM KH_2PO_4, pH 7.4 with NaOH), and then fixed with 4% paraformaldehyde (Alfa Aesar #A11313) in PBS at room temperature for 10 min. After being washed twice with PBS, cells were permeabilized with 0.1% Triton X-100 (Sigma #T8532) in PBS for 10 min. They were then washed with PBS three times and blocked in 1% bovine serum albumin (BSA) (Sigma #SI-A9647) in PBS at room temperature for 1 hr. Cells were incubated in PBS containing 2% BSA and primary antibodies (1:1000, rabbit anti-CSP; Chemicon #AB1576) at 4ºC overnight or at room temperature for 2 hr. After being washed with PBS four times, they were incubated in PBS containing 2% BSA and secondary antibodies conjugated with fluorescent labels (1:1000, Donkey anti-rabbit Alexa Fluor-647; Invitrogen #A31573) at room temperature in the dark for 1 hr. Cells were then washed with PBS five times and coverslips were mounted on slides with Fluoromount G (Electron Microscopy Sciences, #17984-25). Excess liquid was removed, and coverslips were sealed using transparent nail polish. Slides were stored at 4ºC until fluorescent images were acquired by confocal fluorescence microscopy (Leica TCS SP5 Confocal Spectral Microscope Imaging System) under a 100× oil-immersion objective (numerical aperture = 1.40; HCXPL APO CS) with the section thickness about 0.77 μm. Quantification of colocalization was determined with MetaMorph software (Version 7.5, Molecular Devices).

Immunoprecipitation

PC-12 cells [3,62] transfected with WT and mutant CSP constructs were treated with a high-K^+ solution for 15 min and then lysed in the ice-cold RIPA buffer. The soluble fractions were pre-cleared, then immunoprecipitated with anti-CSP antibodies (Chemicon #AB1576) overnight. The complex was incubated with protein G agarose beads for 2h. The immunoprecipitates were washed, boiled with Laemmli sample buffer, and then used for Western blot analysis with primary antibodies [Syx I (Santa Cruz #sc-12736); phospho-PKA substrate (Cell Signaling #9624); CSP (Chemicon #AB1576)].
